# Care at the Very End-of-Life: Dying Cancer Patients and Their Chosen Family’s Needs

**DOI:** 10.3390/cancers9020011

**Published:** 2017-01-24

**Authors:** Katherine Clark

**Affiliations:** 1 Cancer and Palliative Care Network Northern Sydney Local Health District, Royal North Shore Hospital, Pacific Highway, St Leonards, NSW 2065, Australia; Katherine.clark@health.nsw.gov.au; 2 School of Medicine and Public Health, the University of Newcastle, Newcastle, NSW 2981, Australia

**Keywords:** care of the dying, symptom control, care needs

## Abstract

The majority of cancer deaths in countries such as Australia are predictable and most likely to occur in hospital. Despite this, hospitals remain challenged by providing the best care for this fragile cohort, often believing that care with palliative intent at the very end-of-life is not the best approach to care. Given the importance that dying patients place on excellent symptom control, failing to provide good end-of-life care is likely to be contrary to the wishes of the imminently dying patient and their family. This becomes even more significant when the impact of care on the bereavement outcomes of families is considered. Given the rising numbers of predicable hospital deaths, an urgent need to address this exists, requiring health professionals to be cognisant of specific care domains already identified as significant for both patients and those closest to them in knowledge, care and affection. This non-systematic review’s aims are to summarise the symptoms most feared by people imminently facing death which is defined as the terminal phase of life, where death is imminent and likely to occur within hours to days, or very occasionally, weeks. Further, this paper will explore the incidence and management of problems that may affect the dying person which are most feared by their family. The final section of this work includes a brief discussion of the most significant issues that require attention.

## 1. Introduction

The majority of cancer deaths in countries such as Australia are predictable and most likely to occur in hospital [1]. Despite this, hospital-based health care workers do not universally possess the necessary skills and confidence to provide quality health care to dying patients [2]. Rather, health care workers remain challenged by the act of initiating care with palliative intent at the very end-of-life. There remains a belief that delivering palliation rather than curative care is not in the best interests of patients and those important to them [3]. Further, some health professionals are of the belief that the provision of such care might be misconstrued as abandoning the patient and reflective of failure to provide the best care [4].

Given the importance that dying patients place on excellent symptom control, failing to properly address distressing physical symptoms around the time of death is likely to be contrary to the wishes of the imminently dying patient and their chosen family [5]. The impact of this care gap becomes even more significant when considering how care at the time of death affects the bereavement outcomes of friends and families. Recent observations suggest that families affected by the death of a person close to them tend to fare better when they perceived their loved one to have died without discomfort [6,7]. As the total number of hospital based deaths are expected to rise over the next 25 years [1], there is an urgent need to better equip health professionals with sufficient health literacy to best understand how to ensure that this increasingly large cohort of patients receive the best care tailored to their needs.

Providing the best care for dying patients requires health professionals to become more aware of specific care domains previously identified as significant for both patients and their families [8] with families in this context referring to those people who are closest to the patient in knowledge, care and affection. This may include the biological family, the family of acquisition (related by marriage or contract), and the family and friends of choice.

One of the most important issues for patients is their need to be reassured that they will reliably receive expert end-of-life care with the aim of minimising distressing physical symptoms [9]. Such care allows people to live as well as possible despite the fact that death is imminent. Building on this, people with terminal illness have also identified their needs for respectful and compassionate communication to occur between themselves and their health care providers [9]. Attention to these domains engenders trust in health professionals allowing clinicians and patients to work together to establish care goals.

The aim of this non-systematic review is to summarise which symptoms are most feared by people imminently facing death. Differing terminologies are used to describe the time that a person is in the final stages of life both locally in Australia and internationally. The lack of consistency in definitions remains an ongoing source of confusion with terms such as “dying”, “terminal care”, “terminal phase” and “actively dying” or even “passing” tend to be used interchangeably and thus without sufficient descriptive clarity. For this paper, “dying” refers to the terminal phase of life, where death is imminent and likely to occur within hours to days, or very occasionally, weeks.

Further, this paper will explore the incidence and management of problems that may affect the dying person which are most feared by their family. The final section of this work includes a brief discussion of the most significant issues that require attention.

## 2. Symptoms in the Terminal Phase of Life

For some people, the very end of their lives will be complicated by unpleasant and distressing symptoms. A previous systematic review reported the prevalence of symptoms in the final 24 h to last two weeks of life [10]. This review reported data from 12 previously published papers examining the experiences of 2416 patients. The main conclusion was that the symptoms most likely to be bothersome for dying patients were shortness of breath and pain. More recently, the experiences of 18,975 dying patients under the direct or indirect care of hospice/palliative care services were examined with the significant majority of this group (*n* = 14,238) dying of cancer [11]. Nearly 50% (*n* = 5095) of this cohort had pain that was assessed as requiring attention (i.e., pain scores > 3) but only 4.4% were assessed as experiencing severe pain. Other frequently documented problems included fatigue and breathing problems. Far fewer people were assessed as bothered by nausea, loss of appetite or constipation. While these observations suggested that for many people the terminal phase was observed to be a time without distressing symptoms, for some people expert care is required.

## 3. Other Care Needs in the Final Stages of Life

Even aside from physical comfort, patients facing imminent death have identified that other aspects of care are equally important to their wellbeing. This includes the need for excellent culturally-appropriate communication with their health care providers that allows an understanding of their prognosis and what to expect from interventions such as resuscitation [12,13].

Further, people have identified that they do not wish to be overburdened with unnecessary interventions or to be perceived as a burden to others. This latter issue is significant with one review highlighting this as major issue for 19%–65% of terminally ill patients. Poignantly, this has been correlated with patient’s perceptions of loss of dignity, suffering, and a “bad death” [14].

People facing imminent death have also articulated their wishes for place of death. Most important amongst these is the need for privacy and achieving death of in a place of their choice. It is commonly quoted that up to 70% of people have identified that home is their preferred place of death with this figure driving the need to increase community deaths. This has the added benefit of reducing some of the current strains experienced by health systems [1]. However, when the concept of “home” is examined in more detail, it is important to acknowledge that for many, this is an abstract concept and rather than representing a discrete location, home may at times be an expression of the need for those who are important to each other to be close together [15]. Further, achieving a home death is not possible without appropriate resources [16].

## 4. Family’s Needs at the End-of-Life

The chosen family members of dying patients have not unfrequently reported unmet needs regarding their needs for information about their relative’s changing condition, the process of dying, how symptoms would be managed, and what to do at the time of death [17]. Failing to have such conversations may lead to worse psychological outcomes for the dying person’s chosen family members including anxiety and depression [18] and even worse bereavement experience’s [19]. In addition, failing to conduct conversations with families in the timeliest, most culturally appropriate fashion may result in conflict between family members regarding the types of care they perceive their relative should receive. This may lead to families opting for more aggressive care [20].

Families have also identified their own needs for both emotional and practical support over the time their family member was dying. This might be as simple as a comfortable and warm place to sit while being easily able to access food and drinks [21]. After death, self-articulated needs became more complicated with some people requesting support that they felt would allow them to grieve and then move forward [21].

Perhaps the most important aspect of care for the family is to provide the best possible care for the dying person. This is because families are greatly reassured when they perceive their family member had received appropriate attention that ensured the terminal symptoms of pain and breathlessness together with agitation and delirium were as well managed as possible [22].

## 5. The Implications of Failing to Provide Appropriate Care at the End-of-Life

Failing to provide quality care of the imminently dying may unnecessarily expose both the dying patient and their chosen family at risk of a less than optimal experience. Sub-optimal care may result in undue suffering not only for the patient, but their family as well. For example, families whose dying family member received only limited contact with hospice/palliative care are at greater risk of depression compared with others who were linked earlier in the disease trajectory [23]. Further, higher levels of grief, worse bereavement adjustment and depression have been noted when families perceive their dying family member experienced uncontrolled pain or other symptoms [24]. Even aside from grief and sadness, bereaved people are at greater risk of morbidity and even mortality compared to the rest of the community [25]. Grief may be prolonged or result in depression and anxiety. In addition, grief has been associated with increased cortisol secretion which in turn may lead to sleep disturbances, altered immune responses, and prothrombotic responses [26,27].

## 6. Barriers to Quality Care of the Dying

A number of factors explain why imminently dying people are not universally receiving the most appropriate care for their needs. The need to bridge this care gap is increasingly acknowledged by health services with the World Health Organization (WHO) reiterating this is a basic human right [28]. Despite this, many hospital-based health care workers do not feel adequately skilled or resourced to provide such care [29]. Rather, such workers see hospitals as places where sick people receive treatment provided with interventional and curative intent with many health care professionals believing such care is always in the best interest of the patient [30].

The situation is made even more complicated by fact that all too commonly health care workers are not recognizing that their patients are imminently facing the very end of their lives. This is a complicated problem that is likely to arise because of advances in public health and readier availability of more effective treatments. As a result, diseases that may have previously resulted in sudden death are now more likely to be experienced as recurrent episodes of life-threatening exacerbations from which the person recovers [31]. This makes recognition of the final relapse much more challenging, particularly when people have lived for prolonged periods with such life-limiting illnesses.

Many health professionals feel ill-equipped to participate in the necessary discussions around prognosis and end-of-life care. There is a perception that by discussing death and dying with patients and family’s that such negative topics will eliminate hope. However, rather than eliminating hope, such conversations may become opportunities to allow people to articulate what their goals are and by doing so, change the focus as to how health services may support this patient and family to achieve such goals.

## 7. Palpation of Symptoms Identified as Important to Dying Patients

The evidence-base that advises how dying patient’s symptoms are best palliated remains sparse. Conducting research in this cohort requires special attention to ethical and logistical issues that include the vulnerability of dying patients and their families together with their need for privacy. However, this lack of data should make clinicians to be more vigilant and aware of the potential harms associated with prescribing medications for this frail patient cohort. Further, even though rigorous studies that examine care at the very end-of-life are sparse, there is increasingly robust data to inform care of hospice/palliative care patients. The recommendations made here are largely extracted from this evidence-base.

### 7.1. Pain

It is important to monitor pain and treat appropriately which includes the need to undertake regular observations with the aim of monitoring the intensity of the problem and prescribing according to people’s needs rather than diagnosis of dying [32].

Even though the person is dying, thoughtfully assessing the likely cause of pain (or pains) and the experience of this pain for the person is appropriate. This includes considering how this pain is best be addressed and the painful experience modified. For many, this will require strong pain killers such as opioids but in addition, other adjuvant medications or non-pharmacological approaches as recommended in the WHO three step analgesic ladder [33].

When considering how best to manage pain, the same attention to detail is required as when prescribing analgesia at any other stage of life including:

- The person’s past history of pain;
- Previous use of regular or as needed analgesia, including over the counter preparations;
- Most recently available biochemical parameters especially renal and hepatic function;
- A history of allergies or intolerance to opioids and other analgesic agents;
- The most appropriate route of administration that ensures the medication can be safely and effectively administered.

Up to 75% of advanced cancer patients will have sufficiently severe pain that they will require opioid analgesia [34]. These figures make it highly likely that many will enter the terminal phase already receiving opioids. In Australia, people are most commonly switched to a subcutaneous infusion of with morphine or hydromorphone the most common choices. Other opioids including oxycodone, fentanyl and methadone are also available. If necessary, it is important to seek the support of a specialist palliative care service to facilitate the most appropriate choice of medication.

An adjuvant analgesic agent is a medication that has a primary indication for a problem other than pain but possessing some pain-modifying properties in distinct situations [35]. For example, ranitidine is most commonly prescribed for the management of reflux symptoms and peptic ulcer disease. However, a recent randomised control trial supported that the combination of a placebo agent and subcutaneous ranitidine reduced the likelihood of people experiencing abdominal cramping pain from inoperable bowel obstructions compared with octreotide and ranitidine [36].

At this stage of life, one of the challenges when considering how to manage pain with adjuvant agents when people are dying is how such medications can be safely administered when people are no longer able to swallow. Earlier in life, the use of oral or intravenous routes of administration may be considered. However, respecting people’s wishes for the least burdensome approach to care makes this less safe and practical as death approaches. Table 1 lists some easily accessible adjuvant agents that may be safely administered either by a subcutaneous or rectal route of administration when attempting to manage different pain types [37].

### 7.2. Breathlessness

Breathlessness, like pain, is a problem feared by patients facing the end of their lives. Breathlessness is common and is likely to affect at least 24% of cancer patients who do not have predisposing factors such as intrapulmonary pathology or cachexia, and as many as three in five patients with thoracic cancers. The severity of breathlessness is likely to worsen as death approaches with more than 30% of patients likely to experience significant problems in the final stages of life [38].

While there has been much work to advance the palliation of dyspnoea particularly regarding the use of regular low dose morphine, the evidence to support the use of other opioids is sparse [39]. Benzodiazepines are routinely prescribed to address the anxiety that may accompany breathlessness but little evidence supports the efficacy of this recommendation [40]. Oxygen is unlikely to improve breathlessness in this cohort of patients who are not hypoxic but individual patient’s experiences must be considered given recent suggestions that that continuous oxygen might relieve dyspnoea non-hypoxic but breathless patients during exertion [41].

Very commonly, people experience other changes in their breathing patterns at the end-of-life. Such changes include noisy, gurgling respirations presumably due to retained oropharyngeal secretions, as well as Cheyne-Stoke respirations alternating with shallow, rapid respirations. These changes are all very common and are presumed to reflect increasing muscle weakness and dyscoordination with Cheyne-Stokes respirations believed to occur as the result of hyperventilation but prolonged circulation time which results in inability to correct changes acid-base balance [42]. Although numerous clinical guidelines recommend the use of anticholinergic medications to palliate noisy secretions, little evidence supports this practice [43]. Given that these medications may lead to unpleasant complications of dry mouth and agitation, their use is not recommended. More importantly discussion must be had with the family that these changes might occur but are unlikely to be distressing for the dying person [44].

### 7.3. Other Symptoms

Fatigue is likely to affect 50%–90% of cancer patients with few effective pharmacological therapeutic interventions currently available [45]. As disease progresses and people become frailer, the vast majority of cancer patients are likely to experience profound fatigue. Unlike other times of life, this is typically not improved with rest. Even though it is not possible to reverse this problem [46], it is still important to acknowledge this problem with patients [47].

Clinical guidelines often quote nausea and vomiting as distressing problems in the final stages of life. Even though more recent evidence suggests that nausea is less of an issue for dying patients than previously assumed, it is still important to be able to address this problem for should it occur [48]. As at any other time, as far as possible the underlying cause of the problem needs to be considered in order to consider whether tailoring treatment is possible. For example, if a malignant bowel obstruction with cramping pain is suspected, it might be prudent to avoid metoclopramide because of its prokinetic effects on the stomach. Often at the end-of-life, nausea and vomiting are likely to be multifactorial suggesting agents with a broad spectrum of action are likely to be appropriate. The evidence that supports the choice of one agent over another is still limited.

## 8. Addressing Problems Identified as Important to the Families of Dying Patients

### 8.1. Confusion and Agitation

Many patients become confused at the end-of-life. Many families find this distressing particularly when their family member is both confused and agitated. It is estimated that up to 80% of terminally ill patients admitted to a palliative care unit will develop a delirium. For many such patients this is a terminal event [23]. Even a delirium may frequently complicate the end-of-life, the aetiology of this problem remains poorly understood. Further, there is little data available to inform the development of evidence based management guidelines.

At present, an agitated delirium is more likely to be recognised and treated than a pyscho-motor retarded state. Although ideally the management of delirium should include identifying and correcting the most likely cause of the delirium, this is more challenging when a person is imminently dying. This is because a terminal delirium is likely to be multifactorial in origin with numerous investigations at this point not likely to be in the best interest of the dying person.

Most recent evidence suggests that when patients are experiencing mild problems, non-pharmacological approaches to management are preferable [49]. However, when symptoms are more significant, medication may be required. The most commonly recommended antipsychotic remains haloperidol, although risperidone and olanzapine have also been suggested as useful agents at the end life [50]. More data is needed to confirm the most useful agent. Depending upon the degree of agitation, it is not uncommon for a benzodiazepine to be added but given the potential for these medications to worsen the situation, this should be undertaken with care.

### 8.2. Communication

Similar to patient’s expectations, families also rate open and honest communication with health professionals as a very important aspect of good care [51]. Specific issues of importance to the family include ready accessibility to medical staff and participation in formal family conferences. Such conferences are viewed as imperative to allow the family to feel involved in the decision making, understanding the results of investigations and lastly, providing advocacy for the dying person [9].

While such conversations are imperative, many health professionals do not possess the necessary confidence to initiate or participate in these discussions. With support, training and practice, this situation can be improved. Further, there are many guidelines to help support clinicians to better understand how conversations may be framed. It is important to remember that most patients and families prefer to honest and open conversations that allow them to appreciate the situation and plan for the future. By listening and engaging patients and their families it becomes possible to learn that their goals and priories may not include living longer at any cost [52].

Inherent to such conversations is the need to health professionals to be cognisant of any cultural contexts that might impact disclosure and consent preferences of the patient and their family. Specific issues to consider include: the degree to which individual versus family decision making is preferred; whether each family has specific privacy issues; whether there are specific meanings assigned to the disease, its symptoms, medications and nutrition; and whether there are end-of-life rituals that need to be honoured including customs or spiritual and religious preferences [53].

## 9. The Implications of Failing to Provide Appropriate Care at the End-of-Life

Failing to provide quality care of the imminently dying may unnecessarily expose patients and their families to less than optimal experiences around the time the person is dying. Repeatedly, patient reports emphasise that people mostly do not wish to have burdensome and unnecessary interventions. Instead, most people would prefer their symptoms especially pain and breathlessness to be well managed [54]. Failing to provide such care is likely to result not only in due suffering not only for the patient but their family as well [18]. This in turn has implications for the family members’ bereavement [19].

## 10. Opportunities for Improvement

Despite the fact that improving end-of- life care is a priority for many health services, the issues are so complex readily implemented solutions remain elusive. It is insufficient to suggest that improved education of health professionals alone will address this. Rather, system level changes are necessary but even before this there fundamental issues that require discussion.

Many health professionals are confronted by discussions around death and dying. Even though communication skills are increasing considered within undergraduate education in health sciences, ongoing support, education and even credentialing is required to maintain and improve such skills.

The next issue is the ongoing confusion regarding terminology used to describe care at the end-of-life. Words and phrases such as palliative care, terminal care and end-of-life care are used interchangeably to describe care that may be given in the last months of life to care in the last days of life. There is a real need to address this and adopt universal agreement in order to reduce variations in care (Table 2).

Seeking ways to better identify and document the time points when a person’s condition has changed is imperative with the two most important transition points being:

- The change from a person having a chronic complex problem to a chronic progressive problem (entering a palliative phase of care) where life expectancy is now limited by a specific clinical condition.
- When the person is actively dying (entering the terminal phase within a palliative care trajectory) where prognosis is measured in hours to days.

These unique events in disease trajectories are times when patients and their carers need to have informed and compassionate discussion with health professionals regarding their care goals including what they might expect in the future [55,56].

Lastly, agreed quality and safety indicators are required. Despite the fact that a person is dying, equal attention to the quality and safety of care is required as would be expected at any point in a disease trajectory. Agreed quality indicators would allow care audits to be conducted within a quality improvement framework.

## 11. Conclusions

Like any other part of medicine, providing quality of care for the dying requires an understanding of issues likely to impact this specific patient cohort. Furthermore, it is important to seek clarification for each patient and their family the issues that are most important to them. Failing to do this will place people at risk of physical and psychological harms. It is imperative to acknowledge that patients and their families regard good end-of-life care as important to their wellbeing as at any stage of life. This highlights the fact that people wish to live well right up to the time of their death. Even though the available data to inform evidence-based prescribing guidelines is sparse, it remains important to consider to best ways to safely provide care. This patient group is inherently frail with little physiological reserve to tolerate errors. This means that physical harm or psychological insults which in other groups may be well-tolerated, in this group could well be catastrophic: physically, emotionally and psychologically.

## Figures and Tables

**Table 1:** Adjuvant analgesia at the end-of-life [37].

Pain Type	Adjuvant Medications
Musculoskeletal	Paracetamol supp ^a^ 1 g PR ^b^ four times daily Indomethacin supp 100 mg PR daily Dexamethasone inj ^c^ 4 mg subcut ^d^ in the morning
Neuropathic	Dexamethasone inj 4 mg subcut in the morning Clonazepam drops 0.25 mg subling ^e^ at night or every 12 h Clonazepam 0.25 mg inj subcut at night or every 12 h
Solid visceral	Paracetamol supp 1 g PR four times daily Indomethacin supp 100 mg PR daily Dexamethasone inj 4 mg subcut in the morning
Hollow visceral	Hyoscine Butylbromide inj 20 mg subcut four to six times daily increasing up to 120 mg via subcut infusion administered over 24 h Ranitidine inj 200 mg via subcut infusion administered over 24 h

^a^ = suppository; ^b^ = per rectum; ^c^ = injection; ^d^ = subcutaneous; ^e^ = sublingual.

**Table 2:** End-of-life care terminology [55].

**Dying**	The term “dying” refers to the terminal phase of life, where death is imminent and likely to occur within hours to days, or very occasionally, weeks.
**End-of-life**	That part of life where a person is living with, and impaired by, an eventually fatal condition, even if the prognosis is ambiguous or unknown.
